# Advances in Humidity Nanosensors and Their Application: Review

**DOI:** 10.3390/s23042328

**Published:** 2023-02-20

**Authors:** Chin-An Ku, Chen-Kuei Chung

**Affiliations:** Department of Mechanical Engineering, National Cheng Kung University, Tainan 701, Taiwan

**Keywords:** nanosensors, nanostructure, nanomaterials, fabrication, humidity, application, response, sensitivity, nanowires, nanotubes, nanopores, monolayer

## Abstract

As the technology revolution and industrialization have flourished in the last few decades, the development of humidity nanosensors has become more important for the detection and control of humidity in the industry production line, food preservation, chemistry, agriculture and environmental monitoring. The new nanostructured materials and fabrication in nanosensors are linked to better sensor performance, especially for superior humidity sensing, following the intensive research into the design and synthesis of nanomaterials in the last few years. Various nanomaterials, such as ceramics, polymers, semiconductor and sulfide, carbon-based, triboelectrical nanogenerator (TENG), and MXene, have been studied for their potential ability to sense humidity with structures of nanowires, nanotubes, nanopores, and monolayers. These nanosensors have been synthesized via a wide range of processes, including solution synthesis, anodization, physical vapor deposition (PVD), or chemical vapor deposition (CVD). The sensing mechanism, process improvement and nanostructure modulation of different types of materials are mostly inexhaustible, but they are all inseparable from the goals of the effective response, high sensitivity and low response–recovery time of humidity sensors. In this review, we focus on the sensing mechanism of direct and indirect sensing, various fabrication methods, nanomaterial geometry and recent advances in humidity nanosensors. Various types of capacitive, resistive and optical humidity nanosensors are introduced, alongside illustration of the properties and nanostructures of various materials. The similarities and differences of the humidity-sensitive mechanisms of different types of materials are summarized. Applications such as IoT, and the environmental and human-body monitoring of nanosensors are the development trends for futures advancements.

## 1. Introduction

**Figure 1:**
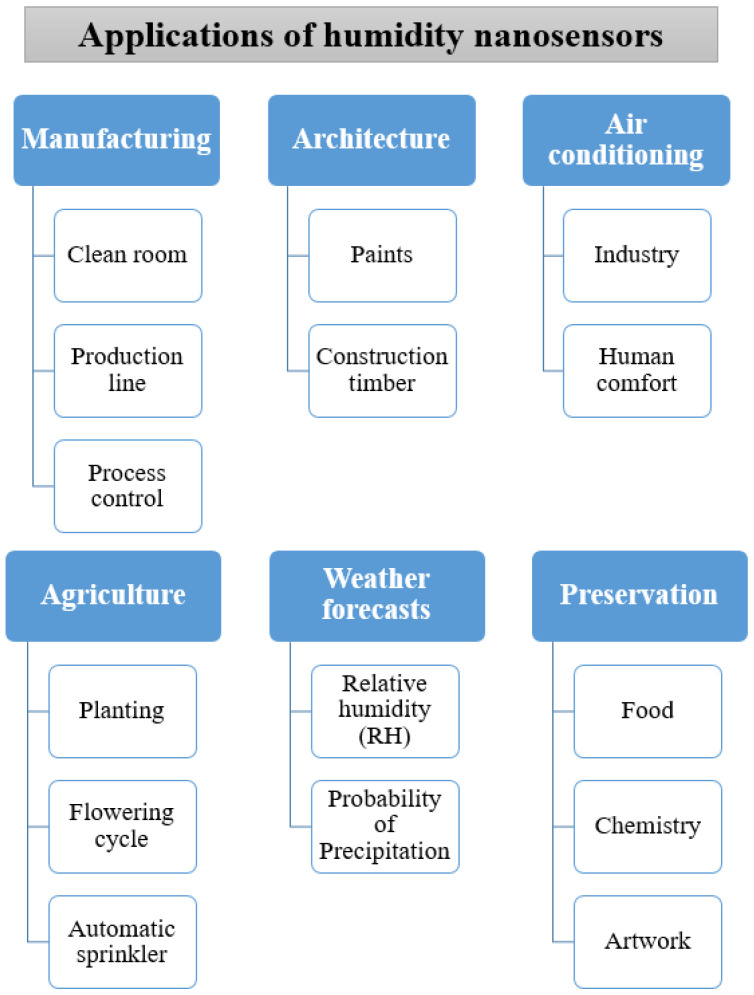
The applications of humidity nanosensors in our daily life.

Relative humidity (RH) is expressed as a percentage, which indicates a present state of absolute humidity relative to the saturation level at a given temperature. As the technology revolution and industrialization have flourished, the measurement of RH has become an important issue in industry, food preservation, chemistry, agriculture, environmental monitoring and our daily life [1,2,3,4,5]. The applications of humidity nanosensors is described in Figure 1, including: (a) manufacturing industry for clean rooms, production lines and process control; (b) architecture for paint and construction timber; (c) air conditioning for industry and human comfort; (d) agriculture for planting, flowering cycle and automatic sprinkler adjustment; (e) weather forecasts for humidity and probability of precipitation reports; and (f) preservation of food, chemistry and artwork. Therefore, accurate and reliable humidity measurement and the performance of humidity sensors for environment monitoring is an important issue in our daily life, and the applications of IoT sensors and human-body monitoring will be a major development trend for advancement in the future.

**Figure 2:**
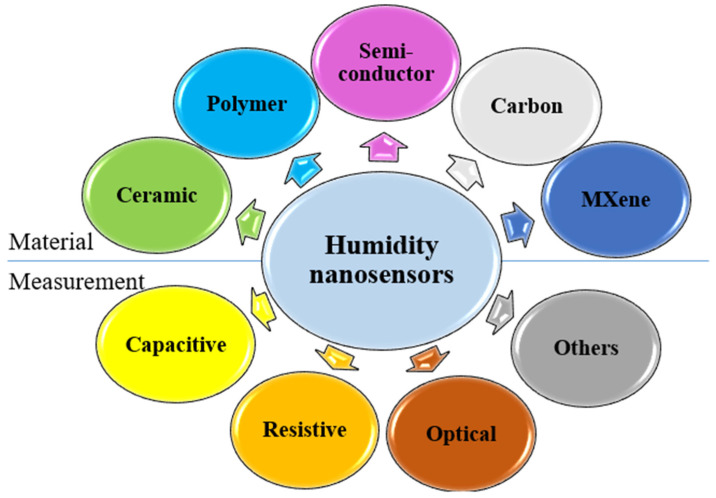
The materials and measurement types of humidity nanosensors.

Some materials and measurement types of humidity nanosensor technologies have been researched in the literature; materials including ceramic [6,7,8,9,10], polymer [11,12,13,14,15,16,17], semiconductor [18,19,20,21,22], carbon-based [23,24,25,26,27,28], and MXene material [29,30] are shown in Figure 2. Generally, the semiconductor and carbon-based humidity sensor can achieve higher sensor response, accompanied by a complex fabrication procedure and longer process time. The ceramic humidity sensors, such as anodic aluminum oxide (AAO), have good thermal stability and wear resistance to overcome severe environments. The polymer-based materials have lower performance in humidity sensing, but it is possible to combine the nanosensor with cellulose for portable devices [31,32,33]. In terms of the humidity measurement types, these can be divided into two categories: electrical [10,26,27,34,35,36,37,38] and optical nanosensors [28]. In electrical sensors, capacitance [10,26,34,35] and resistance [27,36,37,38] measurement types are the most common. Humidity sensors for current [39] measurement are mostly made of semiconductor materials because their resistance will be significantly reduced by humidity increments, resulting in dramatic changes in current. However, with other materials (such as ceramics, polymers), it is difficult to measure current signals due to their high resistance, so capacitance and resistance is still the predominant approach. After the emergence of triboelectric nanogenerators (TENG) for self-powered high-sensitivity sensor applications [40], the measurement method using voltage as an indicator has been extended to humidity sensors. There is an increasing trend of articles using TENG to measure humidity using its voltage variation [41,42,43,44]. Another novel material which has attracted attention for humidity sensing is MXene material [29,30]. The special sensing mechanism of the nanostructure is transformed with high physical and chemical stability, which is worth studying for applications in human health monitoring in our daily life. In addition, optical humidity sensors [45,46,47,48,49,50] can rely on power or wavelength measurement. Optical humidity sensors are mainly made of ceramic or polymer materials, and water-vapor adsorption would change the optical property of these materials, resulting in a color change in the nanosensor. There is also some research focusing on semiconductor-based optical humidity sensors, but electrical properties are still the primary concern for semiconductor materials.

**Figure 3:**
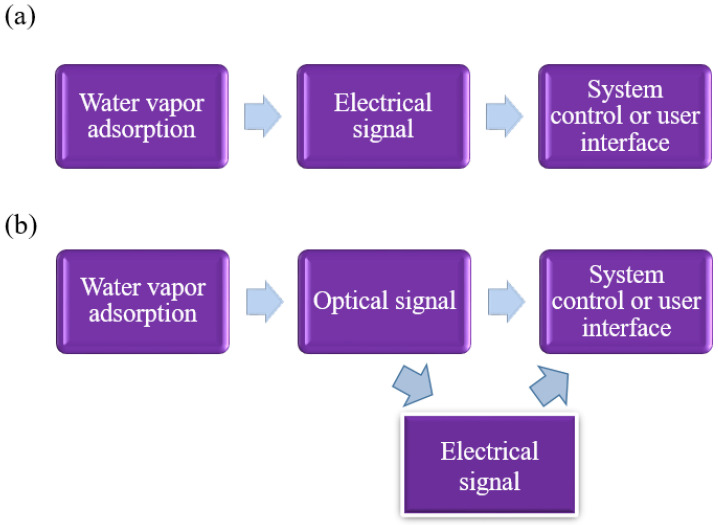
(**a**) The electrical humidity-sensor signal transfer diagram and (**b**) the optical humidity-sensor signal transfer diagram.

The humidity nanosensor system [49] includes the humidity nanosensors, signal transfer and the data-analysis system. The signal change of the humidity sensor comes from the adsorption of water vapor, and then the signal is transmitted to the measurement system or the user interface, as illustrated in Figure 3a. The optical sensor needs to convert the signal into electrical data which can be analyzed by the measuring instrument (Figure 3b), so its cost and system complexity are higher than the electrical sensor, in which most current optical sensor research still employs the naked eye to judge discoloration ability. The recent advances in humidity nanosensors often focus on the development of new functional materials for sensor process enhancements such as low cost and shorter process time, and performance improvement in terms of response, response–recovery time, and stable signal. In this paper, the new nanostructured materials of ceramic, polymer, semiconductor, and carbon-based material and their fabrication processes of solution synthesis, anodization, PVD, or CVD will be reviewed. The sensing mechanism of different materials is discussed, and the sensor measurement types, as well as the performance in terms of response, sensitivity, and response–recovery time will be listed in a table for detailed comparison.

## 2. Materials and Methods for Nanosensor Synthesis

### 2.1. Solution Method

**Figure 4:**
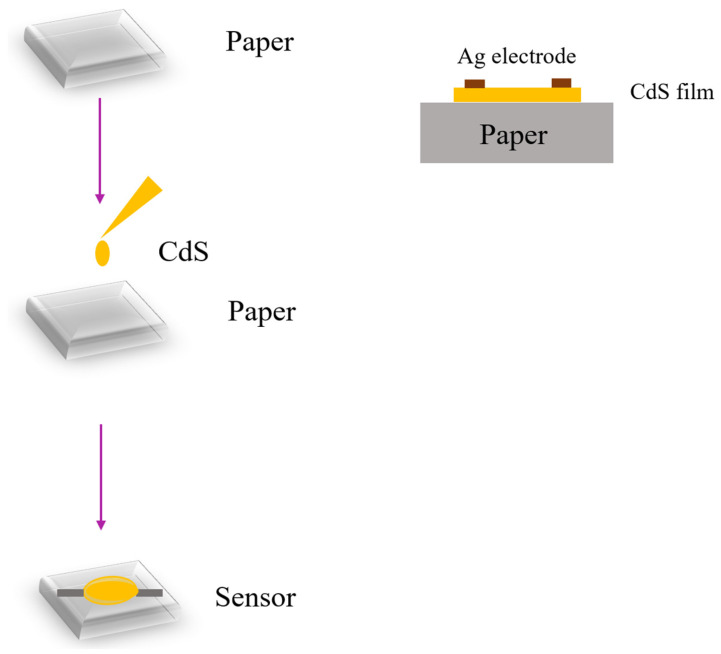
Schematic process flow of CdS semiconductor humidity sensor.

The solution method [51,52,53,54] is the most common fabrication process for functional group synthesis of humidity sensors, especially for polymer, semiconductor, carbon-based, and MXene ones. Solution synthesis can simply be composed of two solutions mixed, or of multiple solutions with the addition of catalysts, sensitizers and dispersants. The solution is usually coated on the sensing substrate using various methods, such as casting [38,52], spin coating [26,51], ink-jet printing [18] or screen printing [54]. For example, Dipankar et al. proposed the CdS nanoparticle-coated paper humidity sensor [38]. The sensing material of CdS nanoparticles was synthesized using the schematic solution method, as shown in Figure 4. The substrate was prepared using filter paper with size of 1 cm × 1 cm, and a cellulose fiber network with 31–38% porosity. The as-prepared CdS nanoparticles with a size of ~3 ± 2 nm were then cast on the substrate. The sensor was then dried for 24 h before the silver Ag electrodes were coated, as depicted in the cross-section schematic diagram in Figure 4. 

**Figure 5:**
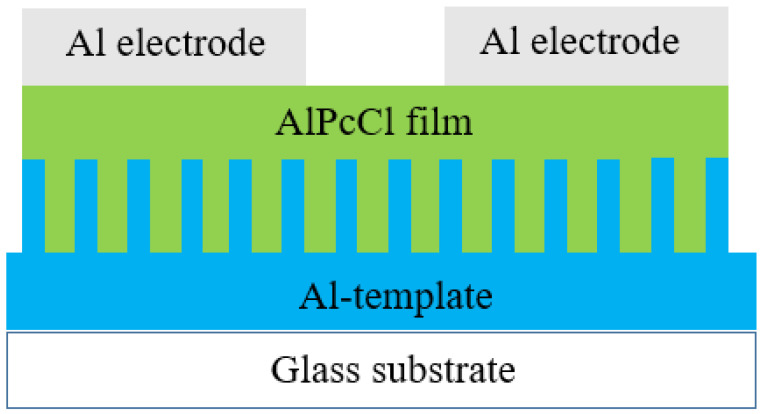
Schematic process flow of AlPcCl-AAO composite humidity sensor with solution method.

Another example was proposed by Ahmad et al.: to develop the organic nanostructure sensing layer on an AAO template for humidity sensing by Aluminum 1,8,15,22-tetrakis 29H, 31H phthalocyanine chloride (chloroaluminum phthalocyanine; AlPcCl) [51]. Two different solutions, by dissolving 5 and 10 mg of AlPcCl powder and 1 mL chloroform (CHCl_3_), were prepared. The nanoporous structures were prepared from a commercially available AAO template with the spin-coating process to from the AlPcCl dielectric thin film, the schematic cross-sectional diagram is shown in Figure 5. In this method, the nano-structure can be controlled through solution concentration and spin rate. The aluminum electrodes were sputtered on the sensor top to form the sensor structure for LCR measurement. In the two papers mentioned above and thoughts on humidity sensors of different materials, higher sensor response is achieved, but this is accompanied by complex solution preparation and longer process times.

### 2.2. Anodization

Anodization is a typical electrochemical deposition technology. Among many humidity-sensor materials, it is mostly used in the preparation of Al_2_O_3_ [51,55,56,57,58,59] and TiO_2_ [60]. This method will be described below in terms of the mechanism of anodic aluminum oxide (AAO) [61,62]. 

**Figure 6:**
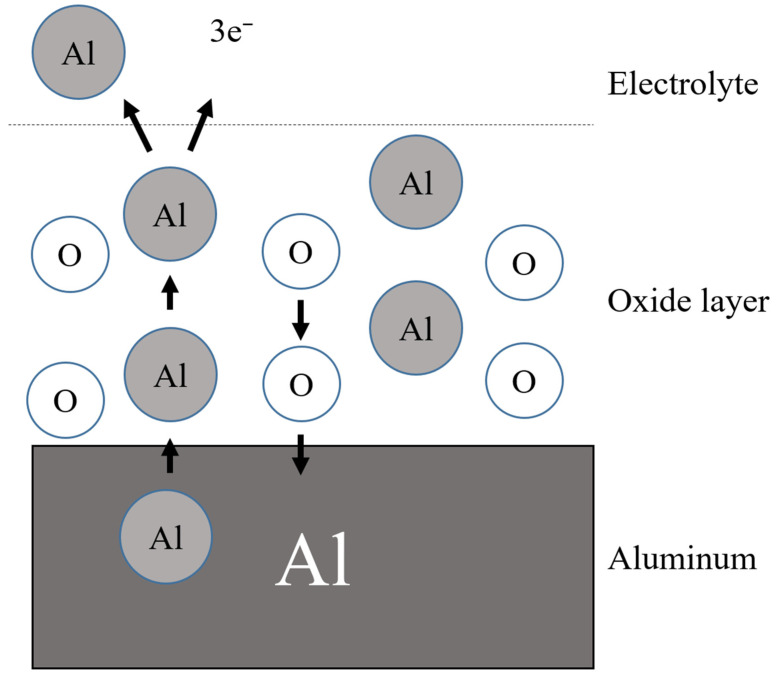
The schematic AAO formation mechanism.

AAO is performed under specific electrochemical conditions. They are mainly created by two chemical equation formulas for the formation of Al_2_O_3_ on Al surfaces and the dissolution of Al^+^ at the barrier layer [61,62], as drawn in Figure 6. The equations are expressed as:

2Al^3+^_(aq)_ + 3OH^−^_(aq)_ → Al_2_O_3(s)_ + 3H^+^_(aq)_ (1)

Al_2_O_3(s)_ + 6H^+^_(aq)_ → 2Al^3+^_(aq)_ + 3H_2_O_(l)_ (2)

**Figure 7:**
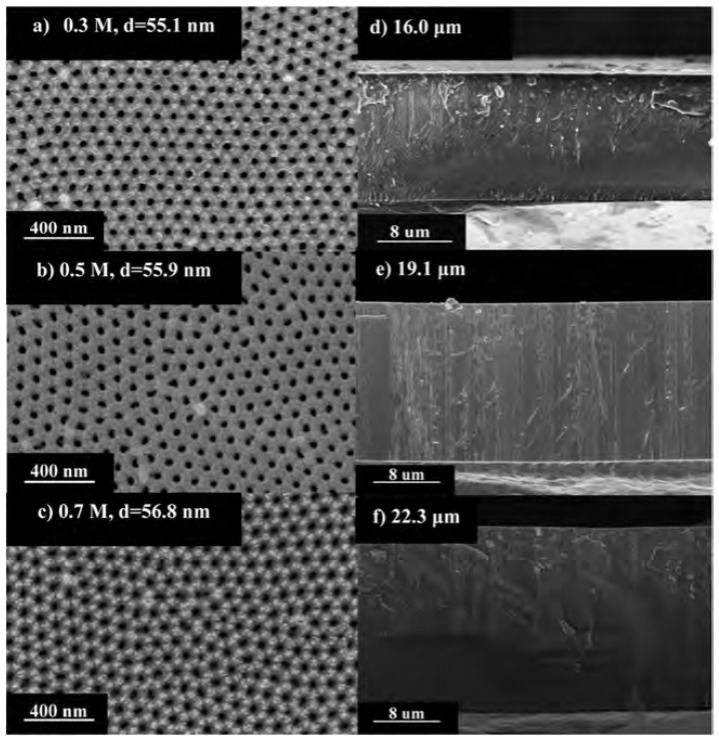
SEM micrographs of the top views and cross sections of the AAO membranes formed through two-step anodization at 40V and 25 °C for 1 h in the oxalic acid solution with concentrations: (**a**,**d**) 0.3 M; (**b**,**e**) 0.5 M; and (**c**,**f**) 0.7 M [62].

During the anodization process, the dissociation degree of water molecules is very low, so the source of oxygen ions is mainly generated by the action of acid radical ions in the electrolyte. When the anions interact with water molecules, the hydrogen ions and hydroxide ions will break to produce hydroxide ions (OH^−^) or oxygen ions (O^2−^). Generally, AAO is anodized in sulfuric acid, oxalic acid, phosphoric acid or chromic acid, with aluminum as the anode and other suitable metals as the cathode. The AAO nanopore structure is modulated by the parameters voltage, electrolyte concentration and time to form various nanostructure. Figure 7 shows the SEM micrographs of AAO nanostructures using different concentrations of oxalic acid [63]. The AAO was performed using a three-electrode electrochemical cell and the potentiostat under two-step direct current anodization at 40V with different concentrations of 0.3, 0.5 and 0.7 M oxalic acid for 1 h, respectively. The pore diameters of AAO analyzed by ImageJ software are 55.1 nm (0.3 M), 55.9 nm (0.5 M) and 56.8 nm (0.7 M), respectively, see Figure 7a–c. The thickness examination at 0.3, 0.5 and 0.7 M of AAO are 16.0 μm, 19.1 μm and 22.3 μm in Figure 7d–f, respectively. The thickness or growth rate of the AAO film increases with the electrolyte concentration due to a general chemical reaction proportional to reactant concentration. The AAO structure grows vertically downward, and the original aluminum metal substrate is beneficial to the parallel capacitor structure. Therefore, most AAO-based humidity sensors are produced with a metal–dielectric–metal structure for capacitance or resistance measurement using the PVD process to contact the electrodes [63,64,65]. On the other hand, some groups used AAO as a template to synthesize nanocomposite films for humidity sensing, such as the AlPcCl-AAO structure in Section 2.1 [51].

### 2.3. PVD

In physical vapor deposition, sputtering [63,64,65,66,67,68,69,70,71] is a method commonly used for various materials of humidity sensors. Sputtering can integrate two completely different materials into one sensing device. At present, there are two processes used: sputtering metal on the sensing material to prepare electrodes, or sputtering the sensing material on the substrate to prepare a humidity-sensing device. In addition, sputtering on flexible substrates such as PET or cellulose is a mainstream process for portable devices.

**Figure 8:**
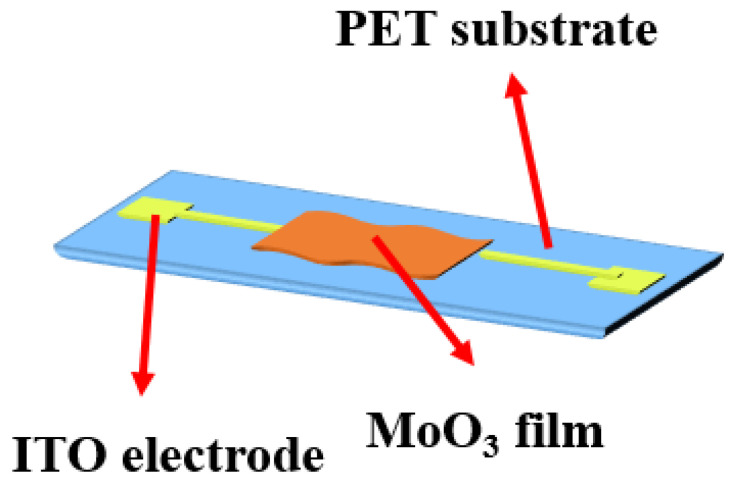
Schematic structure of the flexible humidity nanosensor of MoO_3_ nanosheets on ITO/PET substrates.

Shen et al. proposed a flexible humidity sensor by applying the sputtering method to integrate MoO_3_ nanosheets on an ITO as-deposited PET substrate [66]. First, the MoO_3_ was synthesized using the solution method with stirring and ultrasonicating. For the MoO_3_ humidity-sensing device, the ITO pattern was deposited using photolithography and magnetron sputtering on a flexible PET substrate with a thickness of 50 nm. In addition, a 50 nm Al_2_O_3_ as an insulation layer was then deposited using photolithography and atomic-layer deposition on the bottom electrode. Then, the MoO_3_ solution of the nanosheet structure was spin-coated on the electrode; the sensor structure is drawn in Figure 8.

**Figure 9:**
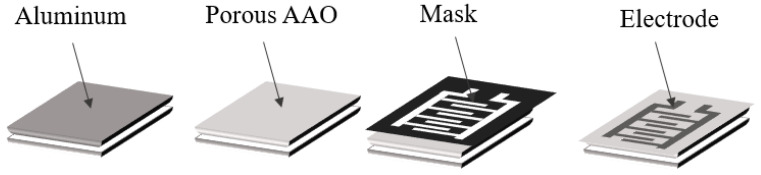
Schematic integration of evaporation and anodization process for AAO-nanosensor fabrication process.

Another PVD process is evaporation, for humidity-sensor preparation. For instance, Balde et al. [35] proposed an evaporation process integrating the anodization process to fabricate a flexible humidity sensor on paper. First, the aluminum metal was evaporated to a thickness of 300 nm on the paper, which was then anodized with phosphoric acid at 100 to 140 V, and then the electrode was evaporated on the AAO with platinum mask for pattern definition. A parallel capacitor structure was formed to integrate the ceramic material onto the polymer-based flexible substrate to create a portable humidity-sensor device. The process flow proposed by Balde et al. [35], with the evaporation of PVD, is depicted in Figure 9.

### 2.4. CVD

Chemical vapor deposition (CVD) is also one of the methods used in the humidity-sensor manufacturing process [72,73,74,75]. Recently, Lee et al. [72] proposed the polymer-based nanomesh humidity sensor for real-time skin humidity monitoring. They used the solution method to synthesize poly(vinyl alcohol) (PVA) using an electrospinning system. Subsequently, Parylene C was deposited using the CVD method until it was 200 nm thick, and then a PVD process was carried out to deposit Au on the top surface to form the sensor structure.

**Figure 10:**
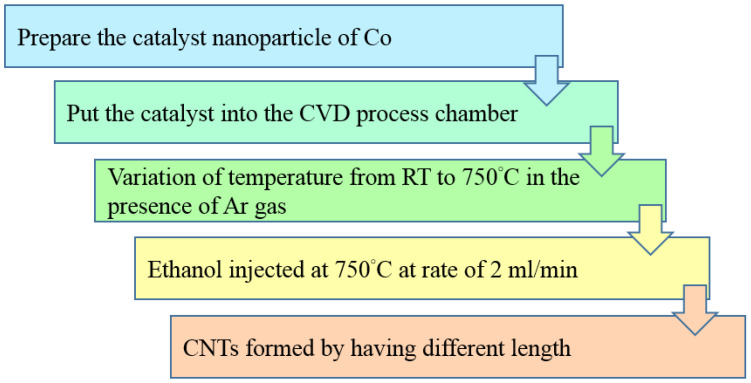
Schematic process flow of MWCNTs formation using CVD method.

Another recent study into using the CVD process to develop humidity sensors was proposed by Yadav et al. [75] using MWCNT for opto-electronic humidity-sensor applications. The experimental process flow is drawn in Figure 10. The catalyst Co nanoparticle was prepared using the sol-gel technique (one of the solution synthesis methods) followed by the supercritical drying process. The 0.5 M solution of cobalt chloride was dissolved in 200 mL ethanol, and stirred for 3 h at 650 rpm, followed by Polyethylene glycol (PEG) and 5 M solution of NaOH drop-added into the solution for catalyst preparation. Second, the catalyst layer prepared was put inside the CVD chamber at a furnace temperature of 750 °C for CNT growth. The CVD process consists of the quartz tube for CNT growth, and was controlled according to the decrease in the quartz tube and temperature. The temperature was maintained at 750 °C for 15 min and then the ethanol was injected into the chamber. Finally, the sensing material of CNTs was formed with different lengths.

**Figure 11:**
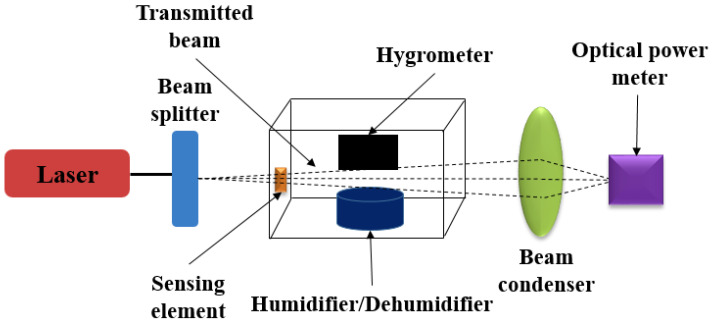
The schematic optical humidity-sensor measurement setup for MWCNT humidity-sensor experiment.

The MWCNT humidity nanosensor measurement setup [75] is redrawn in Figure 11. The system consists of a He–Ne LASER (633 nm) as a light source, with controllable humidity by humidifier/dehumidifier in the chamber, and a hydrometer for humidity monitoring. The sensing element of as-prepared MWCNT was put into the chamber to measure the power change at different humidities through the adjustment of the beam splitter and condenser. The data was collected using the optical power meter system.

## 3. Humidity Nanosensors

### 3.1. Ceramic

The recent innovations, such as new functional materials and fabrication procedures, have significantly accelerated the development of flexible, wearable, and stable electronic humidity sensors. The ceramic sensors, in particular, are in high demand as reliable devices due to their anti-corrosion ability and high thermal stability. The combination of ceramic thin films and polymer-based substrates have already become a new topic of next-generation IoT sensors [6].

**Figure 12:**
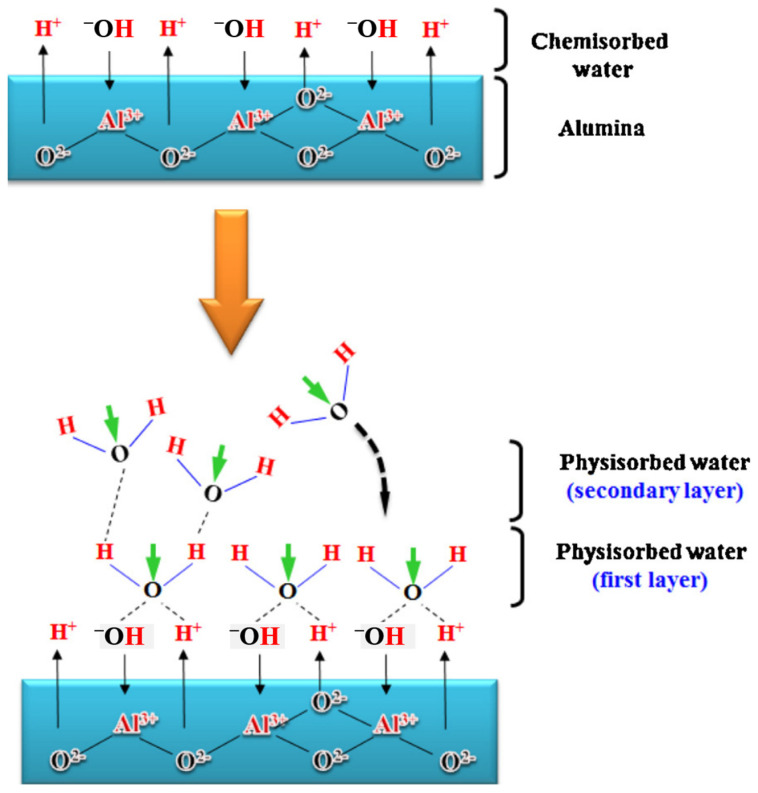
The water-vapor adsorption mechanism in ceramic humidity sensor [77].

**Figure 13:**
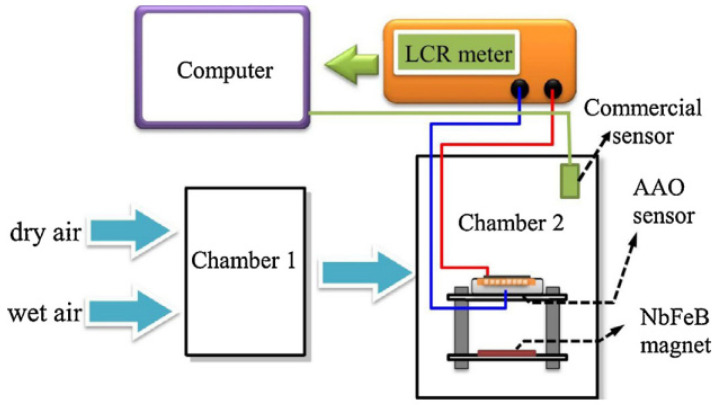
The schematic diagram of electrical humidity-nanosensor measurement system [77].

The ceramic sensor can be approximately divided into two types of electrical sensor: the capacitance type or resistance type, and the optical sensor by spectrum or power measurement. The sensing mechanism of ceramic-based humidity nanosensors is illustrated in Figure 12 with the dielectric from the air of 1 and water molecules of 80. First, Nahar et al. proposed that water molecules initially chemisorbed on the ceramic nanosensors and that there are two hydroxyl groups formed per water molecule to connect the sensor surface [76]. The sensor response changes slightly at this stage at lower relative humidities under 45%. When the RH% rises, the second layer of the physisorbed layer follow water molecules physically bound with the hydroxyl groups of hydrogen bonds that form the first physisorbed layer [76,77]. Hence, it is reasonable to expect the obvious increment in sensor response during the establishment of the first water layer. This phenomenon is the same in both electrical-type and optical-type humidity sensors, but the measurement systems employ slightly different mechanisms. The electrical humidity-nanosensor system includes humidity nanosensors, relative-humidity control chamber and the data-measurement instrument on a computer, as shown in Figure 13. The electrical signal change from the nanosensor is mainly generated by the dielectric difference in capacitance-type sensors and resistivity change in resistance-type sensors. Taking the capacitance-type humidity sensor as an example, the dielectric constant of air and water are 1 and 80, respectively. When the RH% increases, the air will be replaced by water molecules for higher capacitance contribution.

**Figure 14:**
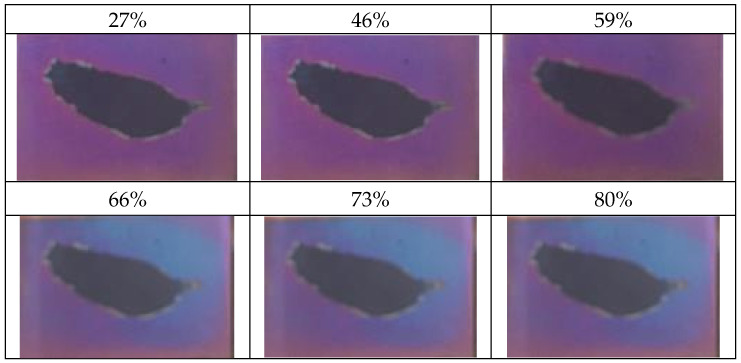
Optical AAO humidity sensor.

On the other hand, the optical nanosensor measurement is based on reflection or transmission for wavelength peaks or power detection. In optical nanosensors, the most famous type is the reflective wavelength-peak measurement. In this case, the optical property change is due to the reflection index (n) change [50]. Take a nanoporous AAO optical humidity sensor as an example, the sensor observed changes from purple to blue at RH% from 27% to 80%, as shown in Figure 14. The structural color difference can be explained using the interference formula:

(3) 2ndcosθ=mλ

where n is the average reflection index of the sensing material, air, and water molecule; d is the film thickness; θ  is the measurement angle; m is the interference stage; and λ  is the wavelength peak. When the water molecule enters the pore of sensor, the average reflection index increases by replacing air with water. This results in the λ  red shift to a higher wavelength, as shown in Figure 14 in the change from purple to blue.

**Figure 15:**
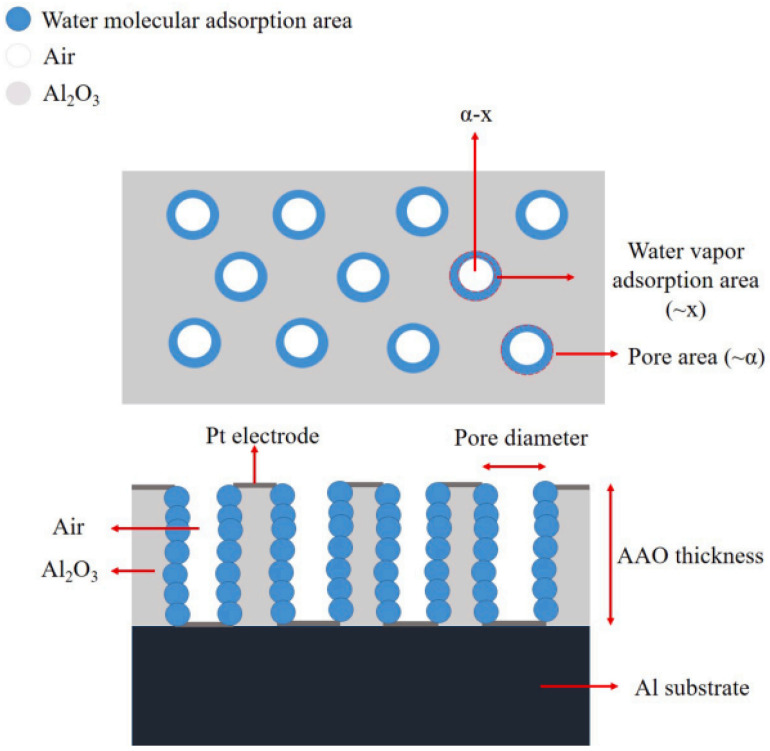
The schematic diagram of AAO capacitor. The water-molecules adsorption ratio is assumed to be x, the porosity is α, and the air area ratio is α–x.

The recent advances in humidity sensors include functional material synthesis and sensor performance enhancement. The improvement in response and sensitivity is reported with high correlation to the specific surface area, because the signal increases with a higher water-molecule adsorption ratio. The AAO humidity nanosensor is a good candidate to prove this assertion, as its pore structure grows vertically downward, as described in Figure 15, a schematic diagram of water-molecule adsorption [59]. The sensor response R is an important indicator of a capacitive sensor and defined as in Formula (4).

(4) $$\mathrm{Response}\;\left(R\right)=\frac{C-C_{0}}{C_{0}}$$

where C and C_0_ are the measured capacitances at RH and dry air (RH of 0%), respectively. To analyze the relationship between R and nanosensor structure, the calculation of capacitance value is an important factor. The capacitance value can be defined with the governing formula:

(5) $$C=\frac{\varepsilon A}{d}$$

where ε, A and d stand for the dielectric constant, area and distance of a parallel capacitance, respectively. The AAO capacitive sensor is a metal–ceramic–metal structure, which can be equivalently regarded as a parallel capacitor in two materials of alumina (C_AAO_) and air in pores (C_pore_) for water molecules (H_2_O) to be diffused and adsorbed onto the pore wall, a, and the equivalent capacitance (C) is described in formula:

(6) $$C\;={\;C}_{\mathrm{AAO}}+{\;C}_{\mathrm{pore}}=\frac{\;[\left(\varepsilon_{\mathrm{pore}}\;A\;\alpha \;+{\;\varepsilon }_{{\mathrm{Al}}_{2}O_{3}}A\;\left(1-\alpha \right)\right]}{d}$$

where ε corresponds to a constant from the different materials, A corresponds to the electrode area, α corresponds to porosity, and d is the thickness of AAO. In addition, the ratio of the water-vapor adsorption area (~x) to the pore area (~α) at a specific humidity is assumed, so that the RH at 0% (C_0_) can be expressed with the Formula (7):

(7) $$C_{0}={\;C}_{\mathrm{AAO}}+{\;C}_{\mathrm{air}}\frac{\;[\left(\varepsilon_{\mathrm{air}}\;A\;\alpha \;+{\;\varepsilon }_{{\mathrm{Al}}_{2}O_{3}}A\;\left(1-\alpha \right)\right]}{d}$$

From Formulas (5) and (7), increasing A and reducing d can benefit the initial capacitance for higher signal intensity. If water molecules diffuse into the nanosensor structure, the ε_(air,H2O)_ is much increased due to the high H_2_O dielectric constant of 80. The ε_air_ and $\varepsilon_{{\mathrm{Al}}_{2}O_{3}}$ are assumed to be 1 and 9.3. Based on Formulas (5) and (7), we can calculate the response, and assume x to be the ratio of water-vapor adsorption under a certain relative humidity. The total capacitance (C_1_) for water-vapor adsorption at a certain humidity level is calculated in the formula:

(8) $$C_{1}={\;C}_{\mathrm{AAO}}+{\;C}_{\mathrm{air}}+{\;C}_{H_{2}O}=\frac{\;\left[\varepsilon_{\mathrm{air}}\;A\;\left(\alpha -x\right)+\varepsilon_{{\mathrm{Al}}_{2}O_{3}}A\;\left(1-\alpha \right)+{\;\varepsilon }_{H_{2}O}\;A\;x\right]}{d}$$

Therefore, the sensor response (R) can be calculated:

(9) $$\mathrm{Response}\;=\frac{C-C_{0}}{C_{0}}=\frac{\;\{\left[\varepsilon_{\mathrm{air}}\;A\;\left(\alpha -x\right)+\varepsilon_{{\mathrm{Al}}_{2}O_{3}}A\;\left(1-\alpha \right)+{\;\varepsilon }_{H_{2}O}\;A\;x\right]-\;[\left(\varepsilon_{\mathrm{air}}\;A\;\alpha \;+\varepsilon_{{\mathrm{Al}}_{2}O_{3}}A\;\left(1-\alpha \right)\right]\}}{\;[\left(\varepsilon_{\mathrm{air}}\;A\;\alpha \;+\varepsilon_{{\mathrm{Al}}_{2}O_{3}}A\;\left(1-\alpha \right)\right]}=\frac{\;\left(\varepsilon_{H_{2}O}\;x\;-{\;\varepsilon }_{\mathrm{air}}\;x\right)}{\left[\varepsilon_{\mathrm{air}}\;\alpha \;+{\;\varepsilon }_{{\mathrm{Al}}_{2}O_{3}}\;\left(1-\alpha \right)\right]}$$

The enhancement of humidity-sensor performance is related to the x and  α. The water-molecules adsorption ratio (x) is linked to the geometry and number of AAO pores concerned with the anodization-voltage-dependent D_p_ and D_int_, which are the parameters for the 2-D top view of AAO. In the formula, both A and d are eliminated, so the water-molecule adsorption ratio (x) is the main factor to judge the sensor response, which is significantly correlated to the specific surface area mentioned. 

The recent advances of several studies into ceramic humidity nanosensors are listed in Table 1 for comparison [10,51,55,56,57,58,59,78,79,80,81]. The ceramic sensors are mainly manufactured using solution methods [10,78,81], anodization [51,55,56,57,58,59] or the PVD [80] process. Due to the poor conductivity of ceramics, the measurement type is divided into two types: capacitance and resistance, or current or voltage types. The sensor performance of the sensitivity in capacitance measurement is defined as:

(10) $$\mathrm{Sensitivity}=\frac{C-C_{0}}{\mathrm{RH}-{\mathrm{RH}}_{\mathrm{initial}}}$$

Compared with response, the sensitivity focuses on the signal change at 1% of RH. In addition, the response and sensitivity in resistive-type sensors are expressed as:

(11) $$\mathrm{Response}\;=\left|\frac{R-R_{0}}{R_{0}}\right|$$

(12) $$\mathrm{Sensitivity}=\left|\frac{R-R_{0}}{\mathrm{RH}-{\mathrm{RH}}_{\mathrm{initial}}}\right|$$

where R and R_0_ stand for the highest and original value of resistance, respectively. In ceramic- and polymer-based sensors, the resistance value at a higher RH% is lower, so the response will not exceed 100%. However, to overcome the limitation of formula calculation, some works proposed the resistive humidity-sensor response formula as:

(13) $$\mathrm{Response}\;=\left|\frac{R-R_{0}}{R}\right|$$

This issue is the factor according to which most papers claimed the capacitance-type nanosensor has better performance. Furthermore, the response, in Table 1 listed below, is about 8000%, which is relatively lower than the semiconductor- and carbon-based humidity sensors. However, this benefits stability in severe environments; in addition, much more research in optical-type humidity sensors, and combinations with polymer-based substrates for flexible devices are discussed in Section 3.2.

### 3.2. Polymers

In recent years, flexible and wearable sensing devices have been of great interest due to their unique characteristics such as portability, endurance, light weight and the combination with the IoT environment-monitoring concept [82,83]. Another superior characteristic of polymer-based humidity sensors is compatibility with other sensing materials. By integrating a sensing material onto a polymer substrate such as cellulose [84,85,86,87,88,89,90] or PET [82,91] using the PVD, CVD or solution methods, it is able to enable the high-strength materials to achieve flexible characteristics. Table 2 lists several different sensing materials with polymer-based substrates [82,84,85,86,87,88,89,90,91]. The semiconductor, graphene oxide and ceramic materials were integrated on PET, cellulose, cellulose nanofibers (CNF), cellulose nanocrystals (CNC), or carboxymethyl cellulose (CMC) for flexible and portable devices. 

**Figure 16:**
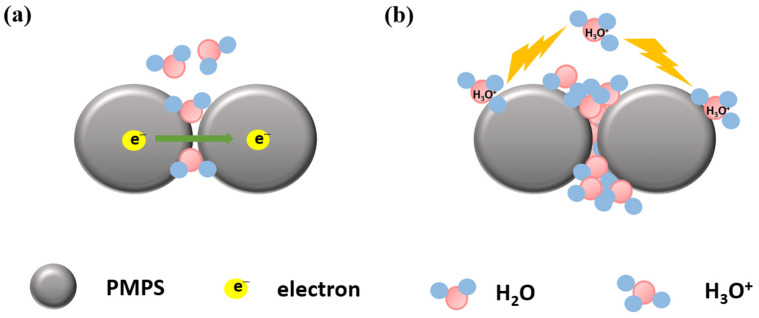
The schematic of polymer humidity-sensing mechanism of PMPS. (**a**) Under low humidity condition and (**b**) high humidity condition.

The sensing mechanism of polymer-based humidity nanosensors is drawn in Figure 16 [92], which is similar to the water adsorption theory in ceramic sensors. The humidity-sensing mechanism of polysquaraine (PMPS) was proposed by Lu et al. [92]. At lower RH values (<54% in this paper), water molecules are adsorbed on the PMPS surfaces but fail to establish a continuous network, so the current cannot pass through by the adsorbed water (Figure 16a), resulting in higher impedance. In Figure 16b, the higher RH values (>54% in this paper) could establish a the water-layer connection between the PMPS beads for higher impedance, and the main charge carrier may be the H_3_O^+^, as depicted. The water-layer connection illustrated here is the same as the physisorbed water-molecule layer mentioned in the sensing mechanism of ceramic nanosensors in Section 3.1. Therefore, we can conclude that the electrical humidity sensors based on ceramic and polymer materials experience similar water-adsorption phenomena.

Several polymer-based humidity sensors are listed in Table 3 for performance comparison [92,93,94,95,96,97]. The measurement types of impedance, resistance and capacitance are commonly used with polymers because of their electricity. The difference in impedance, resistance and capacitance measurement can be expressed using the following equation:

(14) $$Z=R+\frac{1}{\mathrm{jwC}}+\mathrm{jwL}$$

where R corresponds to resistance, jw corresponds to frequency, and L is inductance. When the frequency is smaller, the $\;\frac{1}{\mathrm{jwC}}$ will be larger to dominate the impedance (Z); thus, the frequency is usually set to below 1000 Hz in capacitance measurement. On the contrary, larger frequencies lead the resistance signal to dominate the impedance (Z) result; therefore, resistive-type sensors are usually measured under higher frequencies, of 100,000 Hz. The sensor performance in Table 3 varies from 10% to 855%, which shows relatively lower results in measurement. Although the performance of sensors using polymer as the sensing layer is generally lower in the electrical-measurement type, they are still valued for their excellent compatibility.

In addition, polymer materials play an important role in optical humidity nanosensors because of their unique characteristics such as conformity, light weight and flexibility [45,46,47,48,49,50]. According to measurement methods, optical humidity sensors can be divided into two types: power and wavelength by reflection or transmission. Table 4 lists several polymer-based optical humidity sensors for comparison [45,46,47,50,82,98]. Generally speaking, they are based on the wavelength shift in the reflective spectrum [45,46,47,50,98], or the transmission intensity [82], due to their transparency. Although the color changes in these optical sensors are obvious, they are still not the mainstream, compared with electrical sensing. This is because of the lower response and the necessity to convert the signal before it is read by electronic devices (illustrated in Figure 3). In terms of response, the initial value of the optical nanosensor is mostly in the visible-light (400–800 nm) range, so the wavelength-peak shift is relatively small compared to the initial value, resulting in generally lower sensitivity. In terms of the refractive index, the refractive index of water is 1.33 times that of air. However, the electrical properties, such as dielectric coefficient, have a difference of 80 times between water molecules compared to air. In other words, the optical-properties change is too small compared to the electrical properties of sensor design. Therefore, the optical humidity nanosensors in current development have not yet become a mainstream sensing method. 

### 3.3. Semiconductor

**Figure 17:**
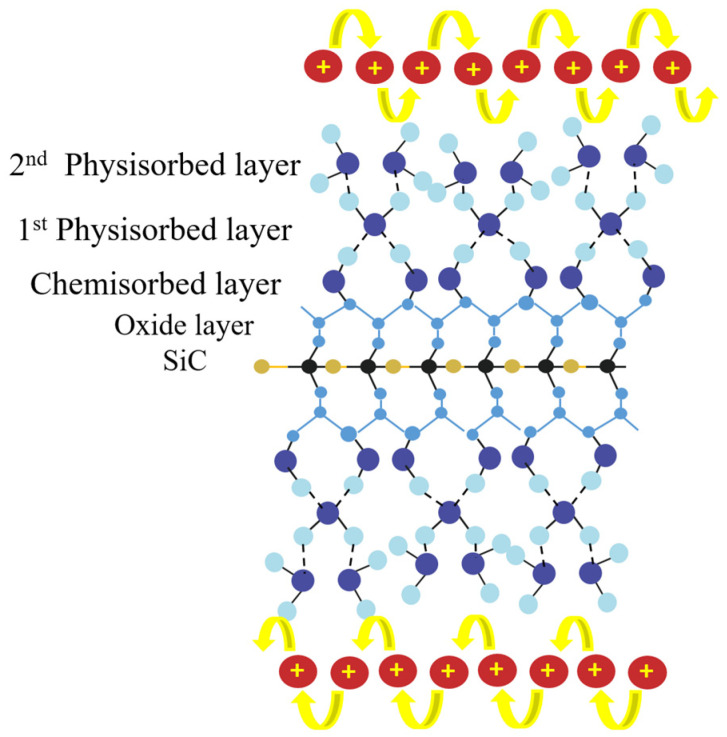
The schematic mechanisms of proton hopping and donor effect in semiconductor-based humidity sensor.

Among all materials, semiconductors are the most widespread material for electrical humidity-sensor fabrication. The sensing mechanism of semiconductor-based humidity sensors, as drawn in Figure 17 [99], is a little different from the ceramics and polymers mentioned in Section 3.1 and Section 3.2. First, conventionally, the “proton hopping” phenomenon from the second physisorbed layer dominates the humidity-sensing mechanism of semiconductor-based humidity sensors [99,100,101]. When the humidity sensor is exposed to water vapor, it initially chemisorbed on the nanosensors and there are two hydroxyl groups formed per water molecule to connect the surface of SiC, as drawn in Figure 17. After the humidity exceeds a certain degree, the water molecules are physisorbed on the chemisorbed layer by hydrogen bonds to form the first physisorbed layer. However, the water molecule on this layer is restricted by double hydrogen bonds, so that it contributes few protons for conducting. As the humidity continues to increase, the second physisorbed water layer is formed by a single hydrogen bond. Due to the weaker force from the single hydrogen bond, water molecules on the second physisorbed water layer contribute a large number of protons by hydronium ions (H_3_O^+^), as illustrated in the Grotthuss chain reaction:

H_2_O + H_3_O^+^ → H_3_O^+^ + H_2_O (15)

The proton is free to transfer along the chain and results in the reduction in the band gap for better electrical conduction [99,100,101]. Another important factor in semiconductor humidity-sensing mechanisms is called the “donor effect” [101]. At lower humidity levels, the surface of SiC adsorbs oxygen, which is easily deionized to be O^−^ or O^2−^ species. These species contribute space for charge accumulation as the depletion layer on the surface. When the humidity increases, the O^−^ or O^2−^ becomes an acceptor for water molecules, which will release the electrons (e^−^) to the depletion layer. Thus, this phenomenon will increase the carrier concentration and affect the band gap of the semiconductor material, resulting in a significant resistance reduction in the sensor. The donor effect happens rapidly and is responsible for the fast response–recovery time of semiconductor humidity sensors. Therefore, the semiconductor electrical humidity sensor is the most widespread to achieve higher sensitivity with a fast response–recovery time. On the other hand, the potential of optical humidity nanosensors is ignored due to its excellent electrical property, so the research into semiconductor-based optical humidity sensors is relatively rare.

Table 5 lists several semiconductor-based humidity sensors for comparison [36,37,38,39,102,103,104,105]. The features of semiconductor humidity sensors are a higher response/sensitivity and lower response–recovery time. In addition to the resistive and capacitance measurement type, the current-type measurement is also a proposed method in semiconductor nansosensor research. For the mechanism discussion in Figure 17, the water-vapor adsorption can significantly affect the band gap and reduce the resistance. Therefore, the current change under a constant applied voltage is obvious. On the other hand, the ceramic- and polymer-based materials are non-conductive, so the current measurement type in humidity sensors is more widespread in semiconductor- and carbon-based materials. In recent years, sulfide-based humidity sensors [106,107,108,109,110,111] are another novel material that research groups are interested in. Most of the sulfide humidity sensors can be categorized as the semiconductor type; for instance, the CdS [36,37,38,111], TiO_2_/PbSnS [106], SnS [107], SnS_2_ [108] or copper, zinc, tin, sulfide (CZTS)-related [109] humidity nanosensors. Generally, the sulfide humidity nanosensors contain the sensing material S or its compounds and have better affinity with water molecules [111]. A 2D material with a high surface-to-volume ratio is of great importance, so there is an opportunity to achieve a higher response [107]. The fabrication method of sulfide-based humidity sensors mainly employs the solution method, which is relatively complicated with requires a longer process time. Some papers are also listed in Table 5 for comparison [107,108,109].

Wang et al. proposed synthesizing the [(Me3)DAB(Me3)] PbI_4_⋅H_2_O with the solution method for humidity-nanosensor fabrication [39]. The sensor was examined at a stable voltage (1 V) to measure the current change at various humidity environments from 10% to 100%. The sensor response was up to 5 orders and estimated with a formula, where I and I_0_ stand for the highest and original value of current, respectively:

(16) $$\mathrm{Response}\;=\left|\frac{I-I_{0}}{I_{0}}\right|$$

The response value can reach to ~1,000,000% in the RH range from 10% to 100%, which is much larger than other works we mentioned except the SnS nanoflake sensor with current-type measurement from 3% to 99% proposed by Tang et al. [107]. The current-type semiconductor-based humidity nanosensors are able to achieve higher responses due to a larger electrical conduction change. In addition, the response value will increase sharply with the measured humidity range, so some researches were able to achieve higher responses from larger RH ranges [39,107,108]. There are also some resistive-type humidity sensors based on different materials with a higher sensor response (>10,000%) [39,105,107]. However, the resistance-response calculation is different for every group, as in Formulas (11) and (13) in Section 3.2. With the RH increases, the resistance will decrease due to the conductance change. In Formula (11), the resistive-type sensor is unable to achieve a higher response than 100%. Taking the CdS/Polyaniline nanosensor proposed by Guo et al. [37] as an example, the resistance varies from 1.8 × 10^5^ KΩ to 90 KΩ at RH% from 11% to 95%. If the Formula (13) is applied to this case, the response can be higher to 200,000%; however, only 100% was calculated using Formula (11). Therefore, some groups define a response-estimation formula in their papers to present the sensor characteristics. For example, Lu et al. [105] defined the sensor response as Formula (17), and the CoTiO_3_/TiO_2_ composite achieved 15723% using Formula (17).

(17) $$S\;=\frac{R_{0}}{R}$$

### 3.4. Carbon-Based

**Figure 18:**
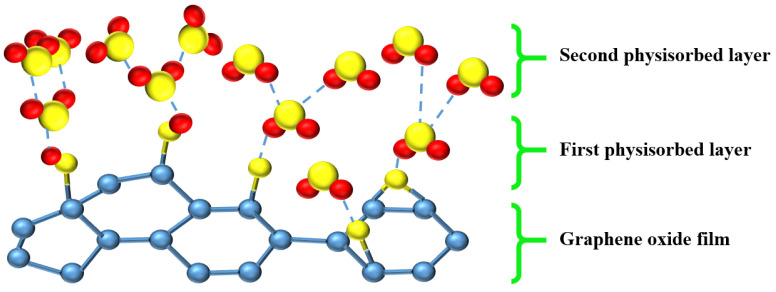
The schematic sensing mechanism of GO.

Recently, two-dimensional (2D) nanomaterials have played an important role in humidity nanosensors due to the larger specific surface area and high carrier mobility. The nanostructures of carbon-based materials, such as grapheme oxide (GO) [88], carbon nanotube (CNT) or carbon nanofiber (CNF) [52,53,112,113,114,115], is an epoch-defining development with a high specific surface area for water-molecule adsorption, and the electrical conductivity brought from free radicals of carbon-based materials makes its humidity-sensing mechanism more interesting to study. The sensing mechanism of carbon-based humidity nanosensors is similar to semiconductor ones, as drawn in Figure 18, from the work of Sun et al. [116]. The water molecules are chemisorbed and physisorbed onto the GO nanostructures. After the humidity increases to exceed a certain degree, the second physisorbed water layer is formed by a single hydrogen bond, and reduces the conductance of humidity sensors. In addition, the “proton hopping” effect occurs on the second physisorbed layer with a significant change in the electricity of the GO. To sum up, four kinds of materials were mentioned regarding their sensing properties, the ceramic- and polymer-based materials are nonconductive, so that the humidity-sensing mechanism is only from the chemisorbed and physisorbed phenomenon of water molecules. On the other hand, the electricity of semiconductor- and carbon-based materials is between conductive and nonconductive; therefore, the band-gap change and proton hopping will occur for significant electricity changes.

Table 6 lists several carbon-based humidity sensors for comparison [24,52,53,88,112,113,114,115,116,117]. The measurement types include impedance, capacitance, current and voltage. As in the discussion of semiconductor sensors, the current detection [115] is easily measured by resistance reduction to several orders. Some groups proposed high response/sensitivity results for their GO/CNT nanosensor [52,116,117] due to the high specific surface area for higher water adsorption ratio. For instance, Chen et al. proposed the highly sensitive sensor based on GO with dispersed multi-walled carbon nanotubes (MWCNTs). The MWCNTs are evenly dispersed in GO solution and restricted in GO layers. The GO and CNTs/CNFs are dispersed in the nanosensor structure, similar to the monolayer for water molecule capture. In addition, the definition of the response calculation affects the results, as mentioned in 3.3 for semiconductor-based nanosensors. Li et al. proposed the graphitic carbon nitride/polyethylene oxide hybrid structure for ultrahigh response to 9,756,300% in impedance. The response calculation is based on Formula (13), due to the lower impedance at higher RH%. Therefore, we can conclude that semiconductor or carbon nanostructured humidity sensors provide a higher sensor response, and must be measured using the resistive or current approaches for larger signal changes. In terms of capacitance, the dielectric constant of water is 80, which is 80 times that of air, but the resistivity can achieve a difference far from 80 times under the influence of band gap; thus, it is reasonable that it achieves the best response/sensitivity. The material with the largest change in surface conductivity must be the first choice, and its specific surface area achieves a higher water-vapor adsorption ratio for better sensor performance.

**Figure 19:**
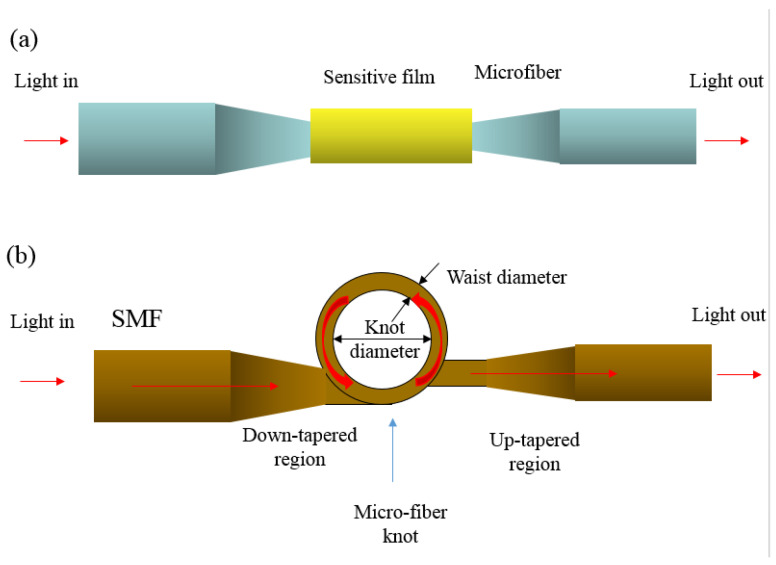
The schematic sensor structure of (**a**) microfiber resonators (MRs) and (**b**) microfiber knot resonators (MKRs). The arrows indicate the light paths.

In terms of the optical sensors made of carbon-based materials, these are mainly proposed with wavelength and power measurement using microfiber knot resonators (MKRs) [118,119], which evolved from microfiber resonators (MRs) by making a tie with the MRs. The MRs humidity nanosensors include polymer, oxides and grapheme oxide as the sensing material, and the microfiber is mainly made of silica [120,121,122]. The difference in MRs and MKRs is the light path change from the knot of the fiber structure, which is drawn in Figure 19a,b, respectively [118,119]. The light passes through the circulating path in the MKRs with a phase shift of 2π, which results in periodic optical resonance in the MKRs. The nanosensor performance is affected by the physical parameters of the waist diameter, knot diameter and the chemical parameter of material characteristic in sensitive film. The recent advances in MKRs development are listed in Table 7 for comparison [118,119,120,121,122]. The silica MKRs are usually applied to MKRs, as optical humidity-sensor research has investigated several sensitive materials to enhance the external perturbations. However, the sensitivity listed in Table 7 is relatively lower compared with the electrical-type humidity sensors, so the MKRs sensors are still primarily used in other fields such as for temperature, strain and pressure sensors.

### 3.5. TENG for Humidity Sensors

**Figure 20:**
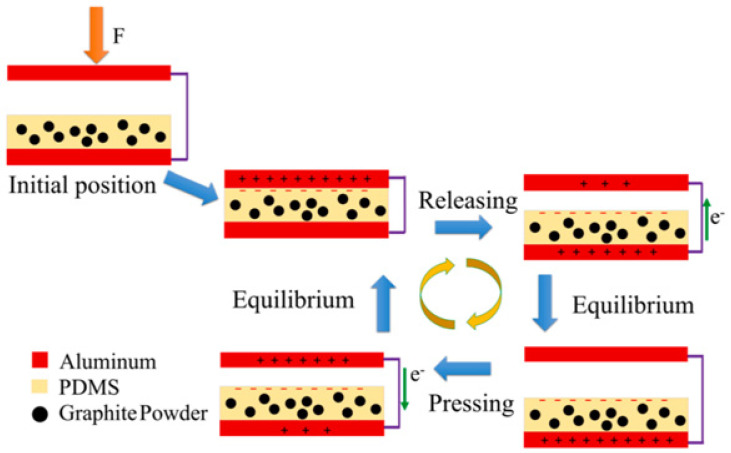
The working principle of TENG from contact and separation mode [129].

To solve the energy crisis and environmental-protection problems of using fossil fuels, energy policies in the new era focus on improving green-energy technology [40,124,125,126]. This has already been a global trend in environmental protection and sustainable energy. Triboelectric nanogenerators (TENGs) are an important solution for harvesting mechanical energy from our daily life. Traditionally, the output performance is dependent on the selected tribo-layers and morphology of TENG; that is, the triboelectricity of the materials is largely determined by the property of the electronic affinity, and the morphology contributed to an effective contact area [40,125,127,128]. Among several tribo-electrical mechanisms for energy harvesting by TENG, the contact and separation mode is the most widespread design for higher efficiency, and is also applied in the humidity-sensing field [41,42,43,44]. Figure 20 shows the working-mechanism diagram of the cycling contact–separation operation of TENGs with positive and negative tribo-layers of aluminum and a graphite-doped PDMS [129]. In the initial position, the two tribo-layers are separated and no charge transfer occurs; then, the two tribo-layer materials are pressed into contact using mechanical force to generate triboelectricity due to the electron affinities of the materials for the electron transfer from the aluminum to the graphite-doped PDMS composite film. At this contact stage, aluminum is positively charged and the graphite-doped PDMS composite film is negatively charged because of their own material tendency. In the releasing stage, with the separation of the triboelectric layers, a potential difference starts to occur between the two electrodes. The electrostatic induction drives the free electrons at the bottom electrode to flow to the upper electrode to balance the potential difference until completed separation. When the tribolayers materials are brought back into contact again, the electrostatic induction motivates the electrons to transfer to the opposite direction again until they are fully contacted.

**Figure 21:**
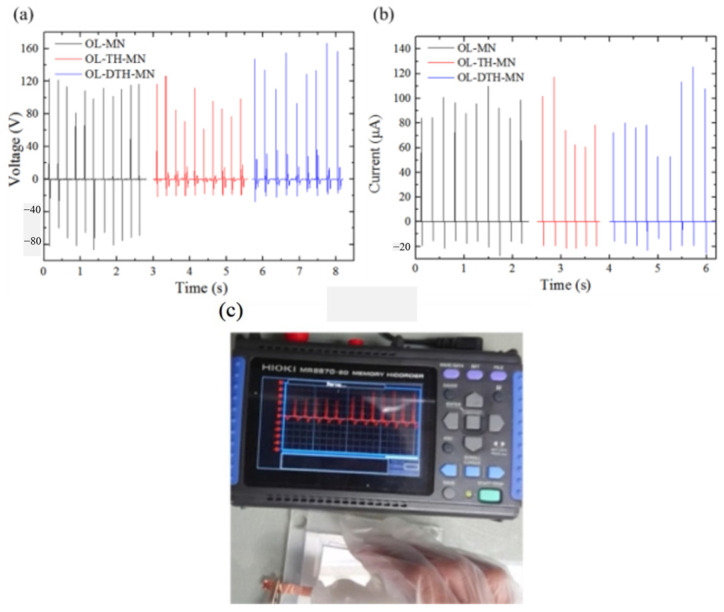
The TENG voltage waveform from cycling contact and separation test, and performance measurement using recorder [130]. (**a**) voltage and (**b**) current was measured of the different TENG during the real-time hand tapping, and (**c**) the image of TENG and measurement device through external force.

The new triboelectric nanogenerator (TENG) technology is successfully used for harvesting wasted energies from motion, sliding, vibration, hydraulic, or air power and has received much attention for effective harvesting energy in numerous practical applications, including consumer electronics, biosensors, pressure sensors, and portable electronic devices. It is an important and a significant issue for TENG devices to develop a cost-effective and rapid processing, to be environmentally friendly, and to develop high-performance technology. The TENG performance-measurement method is shown in Figure 21, with an electrical recorder under the cycling contact and separation mode [130]. The output voltage will change under different conditions, and material and surface morphologies (Figure 21a,b).

In the TENG humidity-sensing field, the mechanism of electrical-signal formation is totally different from the above. The TENG output voltage is mainly affected by the triboelectricity electron transfer from different materials. For the semiconductor- or polymer-based materials we mentioned above, the water-molecule adsorption reduces the resistance on the surface of the sensing material, resulting in an increase in conductance. However, in terms of TENG, the water molecules will restrict the electron transfer and increase the contact resistance from the tribolayers. Therefore, the TENG output voltage will reduce at higher humidities [41,42,44].

Table 8 lists several TENG humidity sensors for comparison. The measurement type is all voltage due to the tribo-electrical characteristics of the TENG. The response value is estimated by Formula (18), where V and V_0_ stand for the highest and original value of voltage, respectively:

(18) $$\mathrm{Response}\;=\left|\frac{V-V_{0}}{V}\right|$$

where V_0_ is the voltage under the lowest humidity measured, and V is the voltage at the highest RH. The influence of the response value of different formulas was discussed in Section 3.2 and Section 3.3. In TENGs, the voltage response is better estimated using Formula (18) than (19), as below, because of the voltage reduction at higher RHs.

(19) $$\mathrm{Response}\;=\left|\frac{V-V_{0}}{V_{0}}\right|$$

In sensor performance discussions, the poly (vinyl alcohol)/MXene nanofber TENG proposed by Zhang et al. achieved a 4000% response and response–recovery time of 0.9/6.3 s. The TENG was synthesized using the solution method for poly (vinyl alcohol)/MXene preparation and MoSe_2_ monolayer from APCVD process for a self-powered TENG. The higher specific surface area of nanofiber can achieve better performance in sensor response.

**Figure 22:**
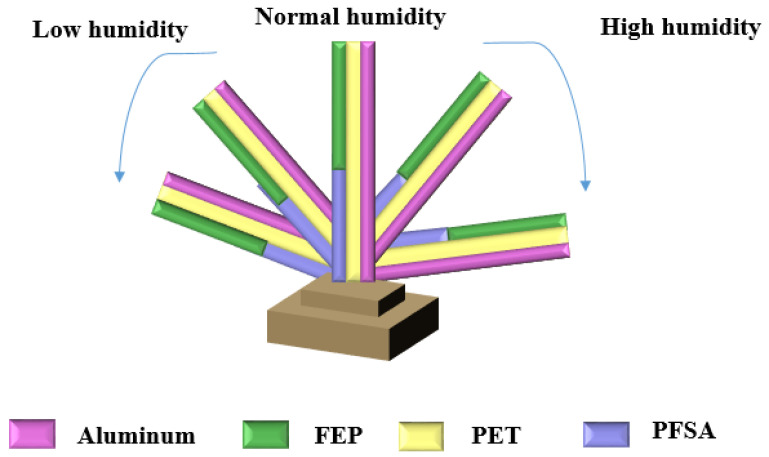
The schematic special humidity actuator by TENG.

Except the electrical humidity nanosensors mentioned above, a special smart deformable TENG was proposed by Chen et al. [43]. The vapor-driven actuator based on perfluorosulfonic acid ionomer (PFSA) was used to detect the RH by automatically bending to different angles. The schematic materials and detailed material structure design of the humidity-responsive TENG actuator is shown in Figure 22, whereas the fluorinated ethylene propylene (FEP) is as a dielectric layer with a negative tendency. With the humidity changes, the TENG can spontaneously bend to different deformation angles and directions through good mechanical strength and flexibility. In addition, the aluminum also works as an electrode to collect the wind or water-drop energy in our environment. 

### 3.6. MXene-Based Humidity Sensor

In recent years, the novel material MXene has been applied to the humidity-sensing field [29,30,132,133,134,135]. MXene is a new two-dimensional (2D) metal carbide and has received much attention due to its high electrical conductivity and chemical stability [29]. It was first proposed by Gogotsi et al. in 2011 using 2D nanosheets synthesized with Ti–C–O–F. It is possible to print MXene through solution synthesis with high precision, and this is performed with high sensitivity in capacitive-type measuring. For instance, Wang and Feng et al. proposed the solution method and electroless deposition for MXene humidity-sensor fabrication [29] with a sensor response of 131.4%. Fabric- or carbon-based substrates are used in combination with an MXene sensing layer for portable or flexible nanosensor devices. In 2022, Han et al. reported the MXene-with-MWCNT sensing device in masks to monitor human breath in our daily lives [30], and achieved a 265% sensor response. In addition, Shen et al. proposed cellulose-fiber substrates for flexible humidity-nanosensor preparation with a 90% response in the same year [134]. Relevant research shows that this is a suitable material for current humidity sensors in the Internet of Things and daily-life monitoring. However, the preparation and synthesis of MXene usually used the HF solution for etching treatment [132,133,134,135]; this is harmful to our environment and dangerous in experiment conduction. The challenge for MXene material is sensor-performance improvement or pollution prevention from the chemical process.

The sensing mechanism of MXene can be divided into two parts. First is the chemisorbed and physisorbed layer of water molecules, which is very similar to ceramic- and polymer-based humidity nanosensors and was discussed in Section 3.1 and Section 3.2. The other is the layer distance in the MXene sensor structure. In lower humidity conditions, the interlayer spacing of MXene nanosheets was mainly formed by the different degrees of the MXene nanostructure. Due to the 2D nanostructure, the interlayer distance between MXene layers would directly influence the electrical property when the structure expanded under high humidity conditions, and the resistance of the sensor showed a more obvious increase [134]. Generally, the electrical signal of resistance will drop when humidity is higher in discussion in Section 3.1, Section 3.2, Section 3.3, Section 3.4 and Section 3.5; thus, this is a major difference between MXene-based nanosensors and other types due to its nanostructure transformation. In addition, the capacitance decreases when humidity become higher, because the distance increases. From Formula (5), the capacitance value is inversely proportional to the thickness (d), so the interlayer distance will cause a lower capacitance signal. This is a major problem to the related research due to the opposite phenomenon of two mechanisms. In the chemisorbed and physisorbed layer of water molecule in higher humidity conditions, the capacitance contribution of water molecule should cause the electrical signal to increase. However, the nanostructure change dominates the capacitance contribution and results in the electrical-signal decrease and shows a lower response. Therefore, the improvement of response or sensitivity is another challenge to the MXene-based nanosensors.

### 3.7. Summary and Future Applications of Humidity Nanosensors

Humidity nanosensors were briefly reviewed and categorized into six types, and the nanosensor performance is affected directly by the material, nanostructure and measurement type and calculation formula. Humidity sensors made of ceramics, polymers, semiconductors, and carbon materials have been developed for a long time, and the research is moving forward with more economical manufacturing processes and better sensor performances. On the other hand, humidity sensors made from TENG and MXene materials have emerged in recent years, and have received more attention. Among the above-mentioned various materials, the semiconductor and carbon humidity sensor can achieve better performance. The sensing mechanism of various materials is related to the adsorption of water vapor and photon hopping, but the adsorption of water molecules significantly changes the electrical properties from semiconductor- and carbon-based sensors. Therefore, in addition to resistance and capacitance, the sensing types also include current and voltage types. The current-type humidity nanosensors are able to achieve a higher response due to a larger electrical conduction change. The response value will increase sharply with the measured humidity range, so some research may be able to reach a higher response from a larger RH range. In addition, the response calculation based on the denominator of the highest value or original data directly affects the response value. For instance, the response calculation based on Formulas (11) and (13) shows significant differences, from 100% to 20,000%, in Section 3.3. On the other hand, the proportion of semiconductor- and carbon-based materials produced by solution synthesis is much higher than for ceramic- or polymer-based nanosensors, and the process tends to be more complicated with a longer process time. Ceramics and polymers are still electrically non-conductive after the adsorption of water vapor, so the response performance is usually not that good; however, this does not mean that they have no research advantages. Ceramic materials have stable physical and chemical properties and can withstand extreme environments, such as high temperatures, compared to semiconductors. Polymer materials have the advantage of flexibility, and a variety of materials are combined with them to form flexible or wearable devices, which is more in-line with future application trends. Among them, TENG is also based on polymer material, but the mechanism is obviously different from the above. The mechanism comes from the triboelectricity of the two materials, so the change in the output voltage measurement is frequently used. MXene is a new material worth studying. The nanostructure of the sensor will transform when humidity changes. However, the contribution of water-vapor adsorption and nanostructural changes is opposite, and HF is often used for etching in the fabrication process. How to improve the process and sensor performance are the challenges of the future. In addition, the sensor performance is linked to a high specific surface area [29,59,107], due to the higher proportion of water-molecule contribution. The electrical signal differences become larger with increasing surface-to-volume ratios [107]. In addition, the formula derivation of total circumference from Chung et al. [59] indicates a similar result. With larger proportions of water-molecule adsorption, a higher sensor response is achieved. Compared with electrical sensors, optical humidity sensors have also been studied extensively, but the observation wavelength of visible light has been limited. Furthermore, the refractive index change from air to water molecules is relatively small compared with the electrical properties, especially the resistivity. It is difficult to surpass the response of electrical sensors. Therefore, how to improve optical sensors is a challenge for the future.

In addition to the traditional applications mentioned in Section 1, humidity sensors are currently developing towards topics such as human-body monitoring, IoT sensors, and environmental monitoring for advancement. For example, the MXene/MWCNT electronic fabric in a mask was proposed by Han et al. [30]. The electronic components, including a microcontroller, analog digital convertor (ADC) module and Bluetooth, were all integrated with sensing layer. From the data feedback, the motion and the moisture from breath, it was possible to perform human health monitoring [30]. Another example is the wearable components combined with polymer materials in a baby-diaper alarm [111]. The resistance was measured and analyzed corresponding with time at different wetness levels in mL for human-body monitoring. The TENG introduced in Section 3.5 is also commonly used in various sensing of the human body and human–machine interface [136], which is a future trend under the development of the IoT sensors. Although the sensor itself can be made non-toxic at present, the comfort of portable devices needs to be further improved in the future. On the other hand, electrical sensors are widely used in the gas sensing [106] of different substances, but water vapor and different gas molecules will affect each other’s signals. For gas sensors, how to prevent moisture interference is an important issue. As for the humidity sensor, how to prevent the interference of other substances is to be considered in the future.

## 4. Conclusions

We briefly reviewed the research progress of the fabrication methods, measurement types, sensing mechanisms and applications of humidity nanosensors in recent years. First, the properties and characteristics of different materials, such as ceramics, polymers, semiconductor, carbon-based, TENG, and MXene humidity nanosensors were summarized. In addition, several fabrication processes, such as solution methods, anodization, PVD and CVD, were described under several conditions for sensor preparation. Dozens of studies are listed in several tables for comparison. The semiconductor- and carbon-based nanosensors synthesized with solution methods are expected to achieve better sensor performance, a higher response of >1,000,000% and shorter response–recovery time, below 1 s.

The recent advance of improving the sensor performance has been linked to high-specific-surface-area [29,59,107] nanostructured materials such as monolayer, nanoflakes or dispersed GO in the last few years. The higher proportion of water-molecule contact, the higher the achieved sensor response. Another important factor to determine the nanosensor performance is the measurement type with proper formula calculation. Generally, the resistive- and current-type measurement systems can amplify the difference in the signal due to the significant change in the resistivity of different RHs, which is much larger than the dielectric constant or refractive index change. Therefore, the semiconductor- and carbon-based electrical nanosensors are the better choice in recent advances for promoting humidity-sensor performance. Although it seems like the best option in the humidity-sensing field, the ceramic- and polymer-based humidity nanosensors are still valuable. Good stability and biocompatibility nanosensors with relatively fast and simple process are reported by ceramic-based studies. In addition, the advantages of being flexible, light weight, and portable are integrated into polymer-based devices for the trend in IoT technology. Furthermore, optical humidity sensing still has a place because the signal can be observed by the naked eye, without requiring electricity. New functional materials, a better fabrication process, improvement in sensor performance, and real-time monitoring from human body will direct the research and development of novel humidity nanosensors to the demand in our daily life under different conditions, which also paves the way for advancements in other applications of IoT, and environmental and human-body monitoring in the future. 

## Figures and Tables

**Table 1:** Ceramic-sensor performance comparison in measurement type, response/sensitivity and response and recovery time.

Sensing Material	Measurement Type	RH Range (% RH)	Response/Sensitivity	Response and Recovery Time	Refs
CaMgFe_1.33_Ti_3_O_12_	Capacitance	33–95	~708% (response)	8.53/11.25 s	[10]
Spin coating polymer material on AAO	Capacitance	20–90	~280% (response)	NA	[51]
AAO on Si	Capacitance	30–90	~4.4% (response)	289/286 s	[55]
AAO	Capacitance	15–80	8000% (response)	45/36 s	[56]
AAO	Capacitance	20–80	5013% (response)	8/9 s	[59]
BaTiO_3_ ink	Capacitance	20–80	575 nF/% RH	37/15 s	[78]
BaTiO_3_-PMMA composite	Capacitance	30–98	1.9pF/%RH	120/60 s	[79]
ZnO nanosheet	Resistance	12–96	220% (response)	600/3 s	[80]
CoCr_2_O_4_	Capacitance	0–95	~350% (response)	NA	[81]

**Table 2:** Humidity sensors using polymer substrates for flexible devices.

Sensing Material	Substrate	Refs
AAO	Paper	[35]
amphiphilic copolymer poly (vinyl alcohol)	PET	[82]
ZnO-cellulose	Cellulose	[84]
CNTs/ZnO/Ag/cellulosic paper	Cellulose	[85]
Graphene-coated cellulosic paper	Cellulose	[86]
CNF with polyethylene glycol (PEG)	Cellulose nanofibers (CNF)	[87]
GO-coated cellulosic paper	Cellulose	[88]
CNC/polyol	Cellulose nanocrystals (CNC)	[89]
CMC/CNTs	Carboxymethyl cellulose (CMC)	[90]
PET	PET	[91]

**Table 3:** The comparison of polymer based electrical type humidity sensors.

Sensing Material	Measurement Type	RH Range (% RH)	Response/Sensitivity	Response and Recovery Time	Refs
polysquaraine	Impedance	33–95	NA	3/16 s	[92]
FeCl_4_/PVDF composite	Resistance	35–90	75%	About 120/180 s	[93]
Keratin bio-composite polymer	Capacitance	16–82	855.66%	30/51 s	[94]
PEDOT/PSS	Resistance	0–28.4	13%	0.63/2.05 s	[95]
polyimide	Capacitance	25–85	16%	NA	[96]
MPOSS-PIL	Impedance	11–95	NA	0.19/0.3 s	[97]

**Table 4:** The polymer-based optical humidity sensors comparison.

Sensing Material	Measurement Type	Wavelength/Intensity Change	Refs
poly(styrene-methyl-methacrylate-acrylic acid)/graphene	Reflective spectrum	About 101 nm	[45]
cellulose nanocrystals/poly(ethylene glycol)/[N-(3-N-benzyl-N,N-dimethylpropyl ammonium chloride)-1,8-naphthalimide]hydrazine	Reflection spectrum	About 164 nm	[46]
poly(diallyldimethylammonium)/poly(styrenesulfonate) polyelectrolyte multilayer	Reflection spectrum	About 129 nm	[47]
konjac glucomannan	Reflection spectrum	About 385 nm	[50]
poly (vinyl alcohol) on PET	Transmission spectrum	About 15% transmission intensity	[82]
cellulose nanocrystals/poly(ethylene glycol)	Reflection spectrum	About 172 nm	[98]

**Table 5:** The semiconductor humidity-nanosensor comparison.

Sensing Material	Measurement Type	RH Range (% RH)	Response/Sensitivity	Response and Recovery Time	Refs
CdS	Resistive	17–85%	NA	~60 s	[36]
CdS/Polyaniline	Resistive	11–95%	NA	~8 s	[37]
CdS	Resistive	5–99%	~60% (response)	~55 s (normal)~3 s (forced)	[38]
SnS nanoflake	Current	3–99%	2,491,000% (response)	6/4 s	[107]
SnS_2_	Resistive	2–99%	154,000% (response)	13.2/0.87 s	[108]
CZTS	Resistive	10–90%	10.77 MΩ/%RH	7.4/58.1 s	[109]
[(Me3)DAB(Me3)] PbI_4_·H_2_O	Current	10–100%	~1,000,000% (response)	NA	[39]
SnO_2_/grapheme oxide	Capacitance	11–97%	1604.89 pF/%RH (sensitivity)	102/6 s	[102]
T3C_2_/polyelectrolyte	Resistive	10−70%	1600% (response)	110/220 ms	[103]
Ti_3_C_2_/TiO_2_ Composite	Capacitance	7−97%	1614 pF/% RH	0.5/2 s	[104]
CoTiO_3_/TiO_2_ Composite	Resistive	11–95%	15,723% (response)	NA	[105]

**Table 6:** The carbon-based humidity nanosensor comparison.

Sensing Material	Measurement Type	RH Range (% RH)	Response/Sensitivity	Response and Recovery Time	Refs
Graphitic carbon nitride	Impedance	11−97	9,756,300% (response)	2.2/3 s	[24]
GO/MWCNT	Capacitance	11−97	7980 pF/% RH (sensitivity)	5/2.5 s	[52]
CNF/CNT	Current	11−95	65% (response)	321/435 s	[53]
Graphene oxide sheets	Capacitance	30−90	5.65 fF/% RH (sensitivity)	NA	[88]
SWCNT	Conductance	10−90	37.5% (response)	6/200 s (10−60% RH)	[112]
MWCNT	Conductance	20−90	61.0% (response)	NA	[113]
Graphite	Voltage	20−70	215% (response)	6/8 min	[114]
GO/MWCNT	Current	11−95	33% (response)	470/500 s	[115]
Graphene oxide	Capacitance	15−95	35,000 pF/% RH (sensitivity)	10.5/41 s	[116]
Carbon nanofiber (CNF)	Capacitance	40−100	3500 %	41/50 s	[117]

**Table 7:** A brief comparison of MKRs humidity sensors.

Sensing Method	Sensitive Material	Sensitivity	Refs
Silica MKRs	MWCNT	1.10 µW/%RH	[75]
Silica MKRs	Silica	0.034 dB/% RH (power)	[120]
Silica MKRs	Graphene Oxide	0.0104 nm/% RH	[121]
Silica MKRs	Polyvinyl Alcohol (PVA)	−1.53 nm/% RH	[122]
MKRs	Ag/TiO_2_	13.4 mW/% RH	[123]

**Table 8:** The comparison of TENG humidity nanosensors.

Sensing Material	Measurement Type	RH Range (% RH)	Response	Response and Recovery Time	Refs
Peanut shell powder (PSP)-based TENG	Voltage	41.5–74.7	About 65%	NA	[41]
Sunflower husk powder based TENG	Voltage	37–89	About 200%	NA	[42]
TiO_2_ based TENG	Voltage	20–84	320%	NA	[44]
Poly(vinyl alcohol)/MXene Nanofber TENG	Voltage	11–97	4000%	0.9/6.3 s	[131]

## Data Availability

Data are the coauthors’ research results and schematic drawings.
